# Efficacy and safety of anakinra in radiation-induced acute pericarditis: a case report

**DOI:** 10.3389/fcvm.2024.1491361

**Published:** 2024-10-14

**Authors:** Ludovico Luca Sicignano, Celeste Ambra Murace, Antonella Palazzo, Elena Verrecchia, Maria Grazia Massaro, Raffaele Manna, Laura Gerardino

**Affiliations:** ^1^ Department of Aging, Neurological, Orthopedic and Head and Neck Sciences, Agostino Gemelli University Polyclinic (IRCCS), Rome, Italy; ^2^ Catholic University of the Sacred Heart, Rome, Italy; ^3^ Comprehensive Cancer Center, Agostino Gemelli University Polyclinic (IRCCS), Rome, Italy

**Keywords:** acute pericarditis, pericardial disease, anakinra, radiation therapy, breast cancer

## Abstract

**Background:**

Acute pericarditis represents an inflammatory disease affecting the pericardial layers. In developed countries more than 80% of pericarditis are defined as idiopathic, less frequently they are secondary to other conditions such as infections, rheumatic or systemic inflammatory diseases, cancer, post–cardiac injury syndromes and radiation therapy (RT).

**Case presentation:**

We reported a case of a patient with acute pericarditis that occurred a few hours after chest radiation therapy, performed for breast cancer. After the failure of first-line therapy, we introduced anakinra, an anti- interleukin-1 (IL-1) agent, observing an immediate clinical and instrumental response.

**Conclusions:**

Our experience highlights the efficacy and safety profile of anakinra in early stages of acute pericarditis with an inflammatory phenotype. To the best of our knowledge, there is no data supporting the use of anakinra in the treatment of RT induced acute pericarditis.

## Introduction

Acute pericarditis represents an inflammatory disease affecting the pericardial layers, with an incidence of 27.7 cases per 100,000 population per year (1). In developed countries more than 80% of pericarditis are defined as idiopathic, less frequently they are secondary to other conditions such as infections, rheumatic or systemic inflammatory diseases, cancer, post–cardiac injury syndromes and radiation therapy (2). In particular, the latter may also represent an obstacle to completing oncological therapy. In those cases, treatment follows recommended guidelines of the European Society of Cardiology, including Nonsteroidal Anti-Inflammatory Drugs (NSAIDs) colchicine and steroids (1). We reported a case of a patient with acute pericarditis that occurred a few hours after chest radiation therapy, performed for breast cancer. After the failure of first-line therapy, we introduced anakinra, an anti-IL1 agent, observing an immediate clinical and instrumental response.

## Case description

**Figure 1:**
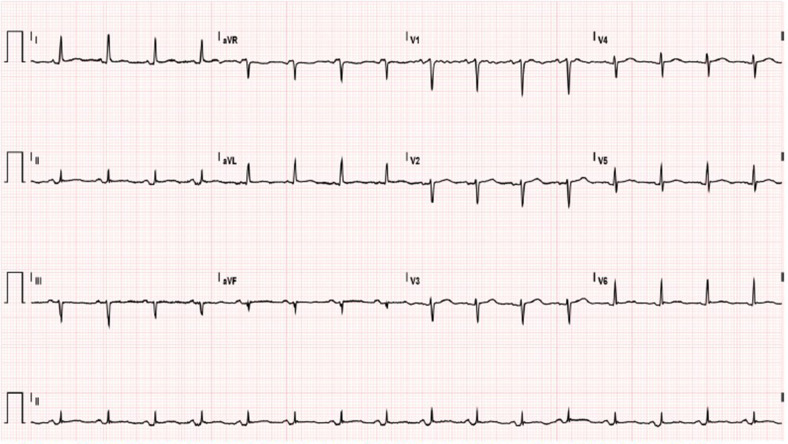
Electrocardiogram shows sinus rhythm with heart rate of 97 bpm, PR depression in DII and a specific ST-T.

A 52-years-old woman, with a recent diagnosis of stage IIIC invasive breast cancer, came to our attention in May 2021 for an acute episode of pleuro-pericarditis occurred during radiation therapy. Her past medical history was significant for depressive and anxious disorder treated with quetiapine and bromazepam. In January 2020 she underwent quadrantectomy with sentinel lymph node biopsy followed by axillary lymph node dissection, which diagnosed pT2 m pN1 (1/11) estrogen and progesterone receptors positive HER2 negative breast cancer. The staging CT scan revealed the presence of a pathological internal mammary lymph node. For this reason, she received anthracycline and taxane based adjuvant chemotherapy, with evidence of radiological partial response on the internal mammary node, followed by letrozole and radiation therapy (RT). In May 2021, after nine doses of RT, she was admitted to hospital for typical chest pain and fever. During the hospitalization, a mild pleuro-pericardial (10 mm) effusion in association with specific electrocardiogram (ECG) abnormalities (Figure 1) and high levels of inflammatory markers [C reactive protein (CRP) 116.7 mg/L, CRP normal value <5 mg/L] were found. Diagnosis of pleuro-pericarditis was made and therapy with ibuprofen 600 mg every 8 h and colchicine 0.5 mg once a day was started. After three days from discharge, she reported a new onset of chest pain and fever and so ibuprofen was uptitrated to 2,400 mg daily. A few days later, she presented to our pericardial clinic complaining about the persistence of symptoms. After ruling out infectious and rheumatic diseases, even if she had received a limited initial radiation dose, we concluded for a diagnosis of suspected radiation-induced acute pericarditis. Therefore, we decided to introduce a different line of therapy and, considering her previous diagnosis of psychiatric disorder, we preferred to use anakinra 100 mg daily over steroids, in agreement with the patient. Thenceforth we observed a rapid resolution of both symptoms and pericardial effusion, with a normalization of inflammatory markers. After one month of treatment, we reduced anakinra to 100 mg every other day and discontinued colchicine, due to neutropenia (neutrophil count 1,200 x 10^9^/L). This dosage was maintained for the next three months, with a good efficacy and tolerance. After one month from the temporarily discontinuation, she restarted and completed the radiation therapy program. Since then, she has attended regular follow-up to our clinic, with progressive tapering of anakinra that was monthly reduced by 100/mg every 7 days till the dosage of 100 mg/week. During the follow up, she reported only one recurrence of pericarditis, which occurred after three months from an attempt of drug suspension. Nevertheless, treatment with anakinra showed good safety and efficacy, being well tolerated without serious adverse effects or severe infections. Moreover, several echocardiographic controls resulted normal, excluding constrictive evolution. Furthermore, the patient continued adjuvant endocrine therapy attending regular follow up visits at the oncologic clinic, without any evidence of cancer relapse.

We report an accurate table of the critical events in the patient’s medical history (Table 1).

**Table 1:** Timeline of critical events.

Day 0	Radiation chest therapy and few hours later sign and symptoms of acute pericarditis. Start treatment with Ibuprofen 1,800 mg/day and colchicine 0.5 mg/day.
Day 5	Up titration of therapy with ibuprofen to 2,400 mg/day plus colchicine 0.5 mg/day because of new onset of pericarditis symptoms.
Day 11	Start of anakinra 100 mg/day due to the persistence of sign and symptoms of pericarditis and new increase of PCR (C-reactive protein) values.
Day 30	Restart of radiation therapy program.
Day 42	Decalage of anakinra therapy to 100 mg every other day and suspension of colchicine because of neutropenia.
10 months later	After a progressive tapering, anakinra is suspended and patient is in good clinical conditions.
13 months later	Single recurrence of pericarditis treated with Ibuprofen and colchicine.
Today	Patient is in good clinical conditions, without any evidence of cancer relapse.

## Discussion

Despite of improved radiation techniques, RT-related side effects are relatively common, including a wide range of pericardial diseases such as acute pericarditis, pericardial effusion, cardiac tamponade, chronic and constrictive pericarditis, pericardial calcification and fibrosis. Acute pericarditis is a potential manifestation of radiation acute toxicity that may occur as early as a few hours after RT administration, with onset of chest pain, fever, and pericardial friction rub. On the contrary, constrictive pericarditis may occur as delayed complication, even up decades after therapy administration, as an expression of chronic toxicity (3). A recent study reported several pathogenic models through which RT may induce cardiac toxicity, including an inflammatory response mediated by pro-inflammatory cytokines, such as tumor necrosis factor-alpha (TNF-α), interleukin-1 (IL-1) and interleukin-6 (IL-6); endothelial dysfunction and fibrosis (4).

In our clinical case, the patient presented acute pericarditis with an inflammatory phenotype characterized by fever, chest pain, high levels of CRP and moderate pleuro-pericardial effusion. After the failure of first-line therapy, we introduced anakinra, an anti-IL-1 agent, observing an immediate clinical and instrumental response. In our decision making, we excluded the possibility to use corticosteroids, avoiding getting worse patient's psychiatric disorder.

By the current ESC guidelines, anakinra is indicated as the possible treatment for colchicine-resistant and steroid dependent recurrent pericarditis (1). To the state of the art, little evidence supports the use of anakinra in acute pericarditis with evidence of safety and efficacy (5, 6).

Our case is interesting for several reasons. First, it represents an example of the efficacy of anakinra in early stages of acute pericarditis with an inflammatory phenotype. Furthermore, to our knowledge, there is no other evidence in favour of its use in RT-acute pericarditis. Anakinra has proven to be able to control the pericardial disease, inducing a prolonged remission and avoiding progression towards constrictive pericarditis. In addition, our experience highlights a good safety profile of the drug, since we had no major side effects and no serious infections occurred. Moreover, it allowed the patient to resume and complete her oncological treatment, without any recurrence of breast cancer, two years after diagnosis.

In conclusion, anakinra may represent a useful option in the treatment of RT-acute pericarditis, although further studies are needed to confirm this data.

## Data Availability

The original contributions presented in the study are included in the article/Supplementary Material, further inquiries can be directed to the corresponding author.
